# Social networks and implementation of evidence-based practices in public youth-serving systems: a mixed-methods study

**DOI:** 10.1186/1748-5908-6-113

**Published:** 2011-09-29

**Authors:** Lawrence A Palinkas, Ian W Holloway, Eric Rice, Dahlia Fuentes, Qiaobing Wu, Patricia Chamberlain

**Affiliations:** 1 School of Social Work, University of Southern California, Los Angeles, CA, USA; 2 Department of Social Work, Chinese University of Hong Kong, Hong Kong, China; 3 Center for Research to Practice, Eugene, OR, USA

## Abstract

**Background:**

The present study examines the structure and operation of social networks of information and advice and their role in making decisions as to whether to adopt new evidence-based practices (EBPs) among agency directors and other program professionals in 12 California counties participating in a large randomized controlled trial.

**Methods:**

Interviews were conducted with 38 directors, assistant directors, and program managers of county probation, mental health, and child welfare departments. Grounded-theory analytic methods were used to identify themes related to EBP adoption and network influences. A web-based survey collected additional quantitative information on members of information and advice networks of study participants. A mixed-methods approach to data analysis was used to create a sociometric data set (n = 176) for examination of associations between advice seeking and network structure.

**Results:**

Systems leaders develop and maintain networks of information and advice based on roles, responsibility, geography, and friendship ties. Networks expose leaders to information about EBPs and opportunities to adopt EBPs; they also influence decisions to adopt EBPs. Individuals in counties at the same stage of implementation accounted for 83% of all network ties. Networks in counties that decided not to implement a specific EBP had no extra-county ties. Implementation of EBPs at the two-year follow-up was associated with the size of county, urban versus rural counties, and in-degree centrality. Collaboration was viewed as critical to implementing EBPs, especially in small, rural counties where agencies have limited resources on their own.

**Conclusions:**

Successful implementation of EBPs requires consideration and utilization of existing social networks of high-status systems leaders that often cut across service organizations and their geographic jurisdictions.

**Trial Registration:**

NCT00880126

## Background

Each year, about 6% of U.S. children and adolescents receive some form of mental health care at an annual cost of more than $11 billion [1]. Despite the increased availability and demand for evidence-based practices (EBPs) for the treatment of youth mental health and behavioral problems [2-5], 90% of publicly funded child welfare, mental health, and juvenile justice systems do not use EBPs [6]. The reasons for this lack of use and the characteristics of systems that predict successful implementation of EBPs remain poorly understood.

Interpersonal contacts within and between organizations and communities are important influences on the adoption of new behaviors [7-9]. Based on Diffusion of Innovations Theory [7] and Social Learning Theory [10], Valente's [11] social-network thresholds model calls for the identification and matching of champions within peer networks that manage organizational agenda setting, change, and evaluation of change (*e.g.*, data collection, evaluation, and feedback). Studies and meta-analyses have also shown that both the influence of trusted others in one's personal network and having access and exposure to external information are important influences on rates of adoption of innovative practices [12-16].

Sociometric techniques for capturing the structure of such networks have been used to study patterns of diffusion of innovations in several arenas, including tobacco prevention programs, contraceptive use and family planning, HIV prevention, and clinical practice guidelines [17]. However, to our knowledge, these techniques have not been used to study the implementation of EBPs in child welfare and child mental health. In addition, these techniques are limited in providing depth of understanding to the process of implementation and to the context in which these influence networks operate. Such depth is usually provided through the application of qualitative methods [18].

Using both quantitative and qualitative data, we sought to accomplish the following: (1) describe the structure and operation of information and advice networks of public youth-serving systems in 12 California counties and (2) determine the influence of these networks in the implementation of an evidence-based intervention designed to reduce placement in group and residential care, juvenile arrest rates, substance abuse, youth violence, and child behavioral and mental health problems.

## Methods

### Setting

The present study uses data from the Cal-40 Study, a clinical trial of an implementation strategy to scale up the use of an EBP for treatment of externalizing behaviors and mental health problems [19,20]. This EBP, called Multidimensional Treatment Foster Care (MTFC) [21], has been shown to reduce out-of-home placement in group and residential care, juvenile arrests, substance abuse, youth violence, pregnancy, and behavioral and emotional problems. The implementation method being tested is the use of community-development teams (CDTs) [22] to scale up MTFC in public youth-serving systems in California; control sites obtain technical assistance for implementing MTFC without the use of CDTs. The Cal-40 study targeted 40 California counties that had not already adopted MTFC. They were matched to form three nearly equivalent groups. The matched groups were then randomly assigned to three sequential cohorts in a wait-list design with staggered start-up timelines (at months 6, 18, or 30). Within each cohort, counties were randomly assigned to CDT or standard implementation conditions, thereby generating six replicate groups of counties, with three assigned to CDT. Across 40 counties, participants are approximately 600 system leaders, agency directors, and practitioners; 400 foster parents; and 900 youth and their families.

Progress toward implementation was assessed by means of a stage-of-implementation checklist (SIC) [19]. Multiple indicators are used to measure both progression through the stage and quality of participation of the individuals involved at each stage. Stages 1-3 track the site's decision to adopt/not adopt MTFC, the feasibility of adoption, their readiness, and the adequacy of their planning to implement. In stage 4, recruitment and training of the MTFC treatment staff (*i.e.*, program supervisor, family therapist, individual therapist, foster parent trainer/recruiter, and behavioral skills trainer) and foster parents are measured. Stage 5 tracks the training and implementation of procedures to measure fidelity of MTFC use. Stage 6 tracks services and consultation to services, including dates of first placement, consult call, clinical meeting, and foster parent meeting. Stage 7 tracks ongoing services, consultation, and fidelity monitoring and how sites use those data to improve adherence. Stage 8 evaluates the site's competency in the domains required for certification as an independent MTFC program.

### Design

We used a mixed-method design that is both exploratory (*i.e.*, by developing the conceptual model of systems leader information and advice networks) and confirmatory (*i.e.*, by testing the conceptual model) [23], achieving three types of integration of quantitative and qualitative data: (1) *convergence*: using both types of data to answer the same question; (2) *complementarity: *using each type of data to answer related questions, where the type of data is specific to the question asked (*e.g.*, using qualitative data to generate hypotheses, provide depth of understanding, and focus on the function and context of social networks and quantitative data to confirm hypotheses, provide breadth of understanding, and focus on social-network structure and predictors of implementation stage); and (3) *expansion*: using one type of data to address questions raised by the use of the other type of data (*e.g.*, using qualitative data to explain results of quantitative analyses) [18].

### Study sample

Participants for the current study included members of the influence networks of the agencies that comprised the first cohort of counties (n = 13) of the Cal-40 Study. As of October 2010, two counties had declined to participate in the study; two counties had reached each of SIC stage 1 (engagement), stage 2 (consideration of feasibility), stage 3 (readiness planning), stage 6 (services and consultation to begin), and stage 7 (ongoing services, model fidelity and feedback); and one county had reached stage 8 (competency/certification/licensure). A purposeful sampling strategy was employed, beginning with directors of the child welfare, mental health, and probation departments of all 13 counties. In some instances, associate directors or senior program managers were recommended by the directors to be interviewed in their place.

Of the 45 administrators from all 13 counties invited to participate, 38 representing 12 counties agreed to do so, yielding a response rate of 84%. Each participant completed a semistructured interview conducted between July and September 2008, with the number of interviews per county ranging from two to six. Twenty-eight participants were interviewed face-to-face; 10 were interviewed by telephone. All those interviewed were then asked to complete a web-based survey to identify individuals on whom they relied for advice regarding EBP implementation. Thirty individuals (86%) of those who participated in semistructured interviews also completed the web-based survey. Data on network ties from the web-based survey were supplemented by additional data provided in participants' qualitative interviews. After complete description of the study to the participants, written informed consent was obtained. The research study was approved by the Institutional Review Board at the University of Southern California.

### Data collection

The semistructured interview centered on knowledge and implementation of MTFC and other EBPs at the county level. Interviewees were asked if they had ever heard of the Cal-40 Study or MTFC and what their motivations were to participate or not participate in the program. Participants were then asked who they had talked to about participation in MTFC or other EBPs; prompts were given to participants as necessary to identify who they talked to, their relationship to that person, their reasons for talking to that person, and the amount of influence that person had on their decision to participate in implementing MTFC or a similar EBP. Then participants were asked about collaborations both within and between county agencies (child welfare, mental health, probation) and the nature of these collaborations. Specifically, participants were asked to identify what made for a successful versus an unsuccessful collaboration. Finally, participants were asked about who usually suggested that their agency take on new programs or initiatives. Probes for influential network actors included agency staff, other agencies, community-based organizations, other county officials, etc.

The web-based survey asked participants to provide general demographic information (*i.e.*, gender, age, number of years in occupation, current position, and time with agency). Per criteria established by Valente and colleagues [15,24], each study participant was asked to identify up to 10 individuals on whom they relied for advice about whether and how to use EBPs for meeting the mental health needs of youth served by their agency.

### Data analysis

A methodology of "Coding Consensus, Co-occurrence, and Comparison" outlined by Willms and colleagues [25] and rooted in grounded theory (*i.e.*, theory derived from data and then illustrated by characteristic examples of data) [26] was used to analyze semistructured interviews. Audio-recorded interviews were transcribed, and lists of codes were developed by each investigator and then matched and integrated into a single codebook. Each text was independently coded by at least two investigators and disagreements in assignment or description of codes was resolved through discussion between investigators and enhanced definition of codes. The final list of codes or codebook, constructed through a consensus of team members, consisted of a numbered list of themes, issues, accounts of behaviors, and opinions that related to organizational and system characteristics that influence implementation of MTFC. The transcripts were then assessed for agreement between the authors on the coding, based on a procedure used in other qualitative studies [27]. Inter-rater reliability was assessed for a subset of pages from 10 transcripts. For all coded text statements, the coders agreed on the codes 91% of the time (range = 88%-94%), indicating good reliability in qualitative research [27]. The computer program NVivo (QSR International, Cambridge, MA, USA) [28] was used for coding and then to generate a series of categories arranged in a treelike structure connecting text segments grouped into separate categories of codes, or "nodes." These nodes and trees were used to further the process of axial and pattern coding to examine the association between different *a priori *and emergent categories.

**Figure 1:**
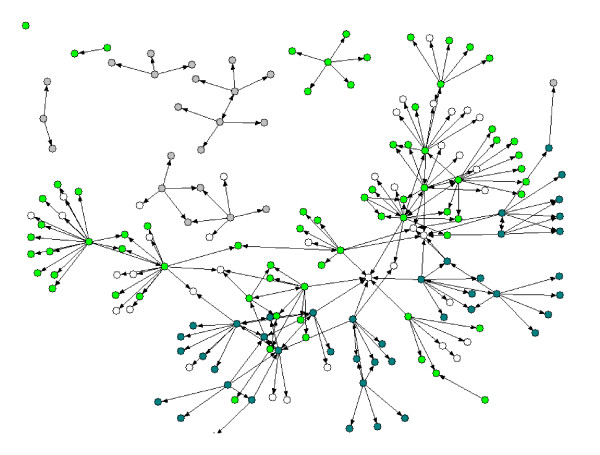
**Evidence-based practice advice networks by implementation stage**. Advice Network Properties. Grey nodes represent individuals who reported on the stage-of-implementation checklist as being in stages 0-1, blue-green nodes represent those in stages 2-6, and bright green nodes those in stages 7-8. White nodes depict individuals about whom insufficient information was obtained to ascertain implementation stage or about whom implementation stage is not relevant, such as individuals who work for the California Institute of Mental Health.

The matrix of ties used to analyze advice networks was constructed from data collected from the web-based survey, supplemented by data collected during the qualitative interviews. The social-network analysis proceeded in three stages: network visualization, structural analysis, and statistical analysis of outcomes. The network visualization was accomplished using NetDraw 2.090 (Analytic Technologies, Lexington, KY, USA). The spring embedder routine was used to generate the network visualizations presented in Figure 1[29].

Structural analyses were then conducted on these network data using Ucinet for Windows, Version 6 (Analytic Technologies, Harvard, MA, USA) [30]. Several network-level measures of structure were assessed, including total number of ties, network size, density (the number of reported links divided by the maximum number of possible links), average distance between nodes, and the number of components (*i.e.*, unique subnetworks). While there are a host of possible metrics from which to choose, we opted for a set of common network metrics in order to provide a descriptive presentation of the network, based on our analysis of data collected from the semistructured interviews. To assess status and interconnectivity within the network, we calculated degree centrality for both incoming ties (being nominated by alters) and outgoing ties (nominating alters). In-degree and out-degree centrality scores assess the relative status of a given node. We also examined several other measures of network status, including between-ness, closeness, and eigenvector centrality. With the exception of eigenvector centrality, these measures were not associated with implementation and were dropped from further analyses. Eigenvector centrality also allows one to examine in-ties relative to out-ties, but in- and out-degree centrality correspond directly to counts of nominations by and toward an actor and, as such, have a straight-forward substantive interpretation, which eigenvectors lack. In-degree and out-degree centrality need not be correlated and, in this network, are not. In-degree captures the status of a node in a network by assessing how frequently that node is nominated by others in the network. This measure reflects how important others in the network perceive a given node to be. Out-degree assesses the involvement of a node in a network by measuring how many others a given node nominates, which may have little to do with how others in that network perceive that node.

Homophily (*i.e.*, likeness between individuals in a network based on specified criteria) data were assessed on three key variables of interest identified during the semistructured interviews: county, agency, and MTFC implementation stage. Homophily scores were created using an algorithm that divided the total number of like ties for that individual based on each of the criteria above by the total ties in that individual's network. Scores ranged between 0 (no homophily) and 1 (perfect homophily). This score can be regarded as the proportion of individuals in a person's network who share a characteristic (*i.e.*, county, agency, implementation stage of MTFC) with that individual. We selected these metrics to assess homophily because our analysis of the qualitative data from the interviews led us to hypothesize that persons in relative proximity to one another (*i.e.*, same agency or same county) would be more apt to communicate. Moreover, we hypothesized that organizations at similar levels of MTFC implementation would be in contact with one another, in part due to their shared stage of adoption.

We used ordinary least-squares multivariate regression models to assess stage of implementation achieved at the two-year follow-up (October 2010) as a function of network- and individual-level properties. Centrality scores calculated in Ucinet were merged with the original data set. We then regressed implementation stage on in-degree and out-degree centrality, adjusting for two county-level dummy variables representing large versus small size and urban versus rural. These analyses were designed to understand how implementation stage varied as a result of position within the sociometric network. Social-network data are derived from nonindependent observations and present a challenge to statistical analysis. To deal with this issue, we employed the most common approach, which is to use a program such as UCINET to generate position-specific variables, which subsequently can be exported to the original individual-level database and analyzed with standard linear models [*e.g.*, [31]]. In cases where the outcomes occur at the level of the tie (not at the level of the node as in the present context), hierarchical linear models with random effects can be employed, which model node-level and tie-level properties as two levels of analysis [*e.g.*, [32]]. As autocorrelation was not found in our data, the issue of independence is primarily a conceptual one.

## Results

Characteristics of study participants are described in Table 1 below. Participants in the study were middle-aged (mean age = 49.38 years), and nearly two-thirds were female (60.5%). Type of agency was evenly divided between child welfare, mental health, and probation. Fourteen of those interviewed were agency directors, 8 were assistant directors, and 16 were program managers. These participants hailed from both large and small counties that were urban and rural and were located throughout the state of California.

**Table 1:** Participant characteristics for social-network data (n = 38)

*Individual characteristics*	
Mean age in years (range)*	49.36 (31 - 63)
Gender	
Male	15 (39.5%)
Female	23 (60.5%)
Agency	
Child Welfare	14 (36.8%)
Mental Health	12 (31.6%)
Probation	12 (31.6%)
Position	
Director	14 (36.8%)
Assistant Director	8 (21.1%)
Program Manager	16 (42.1%)
*County characteristics*	
County size	
Small	20 (52.6%)
Large	18 (47.4%)
Region	
Northern	8 (21.1%)
Bay Area	18 (47.4%)
Central	10 (26.3%)
Southern	2 (5.3%)
Rural county	
Yes	15 (39.5%)
No	23 (60.5%)
*Network characteristics*	
Proportion same county	0.810 (0.226)
Proportion same agency	0.381 (0.266)
Proportion same implementation stage	0.830 (0.223)

*Information on age was missing for eight participants.

### Structure and function of influence networks

Analysis of interview transcripts revealed that systems leaders develop and maintain networks of information and advice according to position in agency (*e.g.*, directors, program managers), responsibility (probation, mental health, child welfare), geography (within a county, neighboring counties), and friendship ties (co-workers, classmates). These networks expose leaders to information about EBPs and opportunities to adopt EBPs and influence decisions to adopt EBPs. This information comes from others within the same county, including supervisors or employees within the same agency, counterparts in other agencies, community-based providers, and community advocates.

Noting both in-county and out-of-county resources for discussing EBPs was common across interviews. Within counties, participants said they drew on advice from individuals in their own agency (although this was not supported to a high degree by network analyses), outside agencies, community-based organizations, and community advocacy organizations. Network members located outside the county included professional organizations like the California Parole Officers Association, the Child Welfare Directors Association, and the California Mental Health Directors Association; intermediaries like the California Institute of Mental Health (CIMH); nonprofit foundations like the Annie E. Casey Foundation and Casey Family Programs; universities; and consultants. Peers from other counties were also an important source of information and advice; however, this occurred more in small rural counties than in large urban counties.

Among the forums for the exchange of information and advice about EBPs are regularly scheduled meetings within the county, region, and state; initiatives that involve contact of systems leaders by CIMH; agency staff; and other county agencies and community-based organizations. One director specifically cited a monthly statewide gathering as a particularly useful venue for gathering information on EBPs:

*"I go monthly to the Children's System of Care meeting in Sacramento. And that's where other people in similar administrative positions to myself who are responsible for children's mental health services, we chew on these kinds of things. We discuss these kinds of things. And, you know, we have presentations, and so forth. So that is my peer group. And that, um, certainly provides a lot of information to me in making decisions."*--Mental Health Department Director

Systems leaders also obtain information and advice on EBPs from counterparts in counties widely regarded for serving as "models" for innovation and EBP implementation, as one agency director noted when asked who she looks to outside her own county:

*"There's a...always [our practice of] checking with Orange County, LA, [when considering adopting a new program]. Although quite big, they do some very progressive things as well. Um, and so you know which counties are kind of doing some leading edge, and, not just leading edge, but that also have uh, the evaluation component of it."*--Chief Probation Officer

Participants described a wide range of advice seeking in qualitative interviews, which included both whether to implement an EBP (MTFC in particular) or a new, innovative program in their county and how to best implement such a program. Social-network survey-based ties between respondents included both types of advice seeking. While some participants in the qualitative interviews simply provided a name of someone who they had contacted about an EBP (or other program), others provided a more elaborate description of the advice-seeking interaction. For example, several participants discussed advice seeking in relation to the cost and feasibility of implementing a particular program; this included instances of where they had decided not to implement a specific program because they had been informed by their counterparts in other counties or directors of community-based organizations within their own county that the cost of implementation would be prohibitive. Others discussed advice seeking related to approaching appropriate community partners for collaboration.

Representations of the influence networks for exchanging information related to EBPs in general are found in Figure 1. Grey nodes represent individuals who reported being in implementation stages 0-1, blue-green nodes represent stages 2-6, and bright green nodes represent stages 7-8. White nodes depict individuals about whom insufficient information was obtained to ascertain implementation stage or about whom implementation stage is not relevant, such as individuals who work for CIMH or other non-county-affiliated organizations. A simple visual inspection of the network diagram reveals that many of the nodes in this network are connected to others in similar implementation stages.

Table 2 provides metrics that help to describe this network. A total of 176 individuals with 233 ties comprised this network. Network density was relatively low; less than one percent of all possible ties among nodes were present. We caution against over-interpreting this metric because mathematically, as network size increases, density decreases [29]. Several other metrics suggested evidence of connectivity. There were eight unique components, that is, "disconnected" sub-networks. One of these components contained 81% of the overall network, while the remaining seven components ranged in size from one to nine individuals. Individuals from 10 of the 12 counties were represented in the largest component, and three counties were each represented in two or more components. Moreover, the average number of ties separating any two individuals in the network was 1.9.

**Table 2:** Network metrics for combined interview and survey network (n = 176)

Metric	Total network
Size	176
Number of ties	223
Density	0.0072
Average distance	1.884
Number of components	8
In-degree centrality	1.27 (0.91)
Out-degree centrality	1.27 (3.05)

The principle of homophily was well supported for both county and implementation stage among members of the original sample. On average, 81% of network ties were among individuals who came from the same county, and 83% of network ties were among individuals who were classified in the same implementation stage as the respondent. Interestingly, only 38% of network ties were among individuals who came from the same county agency as the respondent. Taken together, these results indicate that individuals often rely on others from within their own county for advice on EBPs, although not necessarily individuals from within their agency, and from individuals outside their county. This latter observation was supported by the fact that seven counties had links to one individual who works for the CIMH and is known throughout the state as someone on whom agency directors can rely for information about EBPs.

Implementation stage was also associated with position in the overall advice network. The multivariate regression model presented in Table 3 reveals that county-level and network-position specific variables were important independent correlates of implementation stage. Individuals in large counties, relative to small, reported higher implementation stage, and urban counties, relative to rural, reported higher implementation stage. Increasing in-degree centrality was positively associated with implementation stage at the two-year follow-up, while out-degree centrality was not. These latter results indicate that, adjusting for county-level attributes, being nominated more frequently by others in the network was positively associated with implementation stage two years later, while the number of nominations an individual provided were not associated with implementation stage.

**Table 3:** Regression of implementation stage on centrality, adjusting for county size and urban/rural classification (n = 137)

Variable	B	Standard Error	*t *value	*p *value
In-degree centrality	0.16	0.07	2.26	.03
Out-degree centrality	0.01	0.02	0.61	.54
Large county	0.43	0.14	3.14	.00
Urban county	0.47	0.15	3.24	.00

*Note*: 39 participants are missing from this analysis because their county implementation stage could not be identified or they belonged to an organization for which implementation stage was not appropriate (*e.g.*, California Institute of Mental Health) (*F*(4) = 13.3, *p *< .001; *R*^2 ^= 0.29).

### Collaboration as critical to evidence-based practice implementation

In addition to identifying the potential predictors of implementation stage and supplementing the construction of the social networks, the qualitative analysis of the semistructured interviews identified features of these networks that were critical to the process of EBP implementation. Perhaps the most salient of these features was the role of collaboration within and between counties. Within counties, single agencies often lacked resources to implement EBPs independently and noted that implementation requires good systems partners. In small, rural counties where agencies have limited resources to implement EBPs on their own, agency directors cited a desire to participate in the Cal-40 Study in clusters with neighboring counties.

Poor history of collaboration was often cited as a reason for failure to implement EBPs. The reasons for the lack of collaboration identified by study participants included the following: lack of funding to support a collaboration, different priorities and mandates of the collaborating agencies, different organizational cultures of the collaborating agencies and the lack of understanding of these cultures, and differences in personality and the strained relationships caused by these differences.

Finally, criteria for effective collaborations among agencies in public youth-serving agencies included individuals who can play key roles in the collaborative process, especially agency directors and administrators with knowledge or experience working for another agency who can serve as a collaboration broker or facilitator. For example, one participant cited her varied experience working for multiple agencies as beneficial to understanding complex system interactions, stating, "I fortunately have had the experience of being a probation officer, a social service worker, and a mental health clinician" (Mental Health Department Child/Adolescent Program Chief).

### Role of influence networks in MTFC implementation

These information and advice networks appear to have played an important role in the implementation of MTFC among the first cohort of counties participating in the Cal-40 Study. For those who had agreed to participate or were considering participation at the time they were interviewed, information about MTFC and the Cal-40 Study was obtained from presentations given by CIMH representatives at state or regional meetings, direct contact by CIMH with county agency directors, direct contact by other agency directors within the county, or staff within the agency:

"*It came to my attention two different ways. I started hearing some discussion about it at the small county association meetings, which is a break off of the full body county Mental Health Directors Association. And I heard it from one or two of my peers... Then, the newest program manager brought it to my attention. And I think she found it on the CIMH website..."*--Mental Health Program Director

Only one of the seven systems leaders interviewed from the three counties that had either decided not to participate in the Cal-40 Study or had not advanced beyond stage 1 had received any information about MTFC or the Cal-40 Study.

## Discussion

The results of this study suggest that the structure and operation of social networks--specifically, higher in-degree centrality of network members, as well as network context, reflected in the size of county and whether it was predominately urban or rural--are central to implementation of EBPs. Further, social networks influence the implementation process through two mechanisms, development and operation of successful collaborations and acquisition of information and support related to EBPs. Although many factors influence the diffusion of EBPs, researchers have consistently found that interpersonal contacts within and between organizations and communities are important influences on the adoption of new behaviors [7,8,33-36].

In this study, the majority of network ties occurred within the same county and same implementation stage. This is understandable given that both randomization and use of the SIC measurement in the Cal-40 Study occurred at the county level [19]. However, only a little over one-third of network ties existed among individuals in the same agency. This could be accounted for, in part, by the Cal-40 Study requirements that at least two of the three agencies in a county had to agree to participate, one of which had to be the mental health agency, in order to enroll in the study [19]. The results also supported the importance of collaboration between agencies. This was reflected in the number of ties among individuals representing different agencies in the same county and the qualitative data highlighting the importance of collaboration for EBP implementation, especially in resource-poor rural counties, even when participation of more than one agency is not a requirement for implementation of a specific EBP.

The results of this study also help us to understand the context in which these networks influence the implementation of EBPs and how differences in context, like the size of a county or the structure of personal networks, can influence whether or not EBPs are adopted by public youth-serving agencies. Our results suggest that characteristics of the county and in-degree centrality are associated with EBP implementation stage. Characteristics of the county include its size and urban/rural status. In our sample, larger, urban counties were classified in a higher implementation stage than their smaller, rural counterparts. A similar association between county size and days to consent to participate in the Cal-40 Study in all three California cohorts was reported by Wang and colleagues [20]. Analysis of qualitative interviews with systems leaders found that small, rural counties often lack the resources to implement innovative practices on their own due to a limited supply of qualified staff, funding, and available clients. The two counties that declined to participate in the Cal-40 Study were small, rural counties possessing networks that were also small and lacking ties to other networks that had decided to participate in the study and were proceeding with MTFC implementation. These findings highlight the importance of networks involving ties to counties with resources or the pooling of resources via existing networks. These networks also exposed agency directors and senior administrators to information about and opportunities to implement EBPs, which, in turn, influenced decisions about whether or not to implement these practices.

However, we also found that MTFC implementation stage at the two-year follow-up was associated with position in the overall advice networks at baseline. Higher-status individuals, measured by in-degree centrality, were more likely to work in counties that achieved a higher stage of implementation two years later. These individuals were nominated by others as a source of information and advice about EBPs and innovative programs in general. The central position of these individuals in influence networks makes sense since systems leaders would be inclined to seek information and advice from someone who had experience and was successful in implementing such practices. These findings are also consistent with Valente and colleagues' findings of the association between the presence of opinion leaders in one's social networks and rates of adoption of innovative practices [12-16].

Not all opinion leaders need have a high degree of centrality; in some cases, opinion leaders are persons who bridge different social networks, and their position as a bridging tie facilitates their success in bringing new practices from one network to another [37]. There are several nodes in this network whose structural position could allow for such bridging between sub-networks. Further, although our results point to an association between stage of implementation and in-degree centrality but not out-degree centrality, it is possible that these two forms of status operate differently at different stages of implementation, with the former being more important in the earlier stages and the latter being more important in subsequent stages.

Our study results also provide an indication of how influence networks operate to implement EBPs. The semistructured interviews provided numerous instances of exchange of information within agencies, within counties, and across counties. This exchange usually occurred through regularly scheduled meetings or conferences, through a search for information concerning the EBP by the systems leader, or through dissemination efforts of intermediary organizations like CIMH. Influence networks also operate to implement EBPs by sharing resources, which include funding, staffing, or consumers. This sharing is easier in large counties because agencies in these counties possess more resources than similar agencies in small counties. However, the existence of subgroups or cliques may preclude sharing due to competition for the same resources. In smaller, rural counties, on the other hand, resources are often shared between agencies in the same counties or with agencies in neighboring counties because the individual agency frequently lacks the capital, staffing, or consumer demand necessary to initiate or sustain implementation efforts.

Implementation was also associated with greater connectivity across counties. Counties who declined to participate or did not advance beyond stage 1 had no ties or links outside the county. In contrast, counties that had achieved stage 6 or higher were all linked to CIMH, a primary source of information on EBPs in the state. Most of the network links to CIMH were with county mental health agency leaders, which is understandable given the involvement of CIMH in regularly scheduled meetings of the California Mental Health Directors Association and with county Chief Probation Officers, which can also be explained by the fact that the key CIMH "node" was a former county chief probation officer.

One of the conclusions to be drawn from this research is that implementation strategies should be designed to either build influence networks or capitalize on existing networks. The CDT approach being tested in the parent study is designed to build social networks that offer support to network members in implementing EBPs. Other strategies with a similar aim include the Institute for Healthcare Innovation's Breakthrough Series collaborative [38]. Dissemination efforts can and should make use of existing networks. For instance, as revealed in the interviews with systems leaders in this study, networks provide access to opportunities to observe firsthand the implementation and effectiveness of EBPs in systems that are regarded as models or early adopters. Strategies for implementation should strive to create partnerships between agencies within counties that serve the same target population and build influence networks across counties, thereby enabling systems leaders in agencies based in small rural counties or possessing small influence networks to acquire more information and resources from leaders in agencies based in large urban counties.

There are several limitations to our study that deserve mention. First, this investigation was conducted during the initial or first steps of EBP implementation with a small number of counties. Although our findings suggest that there will be changes in patterns and processes of implementation over time, we were primarily interested in examining networks at the initial stages of the implementation process and then determining whether these "baseline" networks could predict the implementation trajectory over a two-year period. Second, systems leaders who participated in interviews at this stage of the Cal-40 Study represent almost all of the first cohort but may not represent the broader population of systems leaders participating in other cohorts, much less the broader population of systems leaders engaged in child and adolescent mental health services. Thus, the results obtained thus far may not generalize to either population, although cohort 1 counties were selected through a process of randomization and thus should be representative of all 40 counties participating in the parent study. Third, the 176-member network was constructed based on information from 38 interviewees who were not asked to provide information on sociodemographic and occupational characteristics on those they nominated. Consequently, we lacked individual-level measures on some of the nodes who were not directly interviewed, thereby limiting our statistical power to examine the influence of such characteristics as predictors of network structure or implementation stage. Finally, both collection and interpretation of qualitative data is susceptible to subjective bias and preconceived ideas of the investigators. However, the use of multiple observers as well as multiple sources of data to achieve "triangulation" [39] should minimize such bias.

## Conclusions

Despite these limitations, the results of this study suggest that social networking is central to implementation of EBPs through two mechanisms: development and operation of successful collaborations and acquisition of information and support related to EBPs. The most influential networks appear to be those that extend beyond service-system jurisdictions. This study helps us to understand the context in which these networks influence EBP implementation and how differences in context of personal networks can influence whether or not EBPs are adopted by public youth-serving agencies. It also helps to inform the design of implementation strategies that either build influence networks or capitalize on existing networks.

## Competing interests

PC is a partner in Treatment Foster Care Consultants, Inc., a company that provides consultation to systems and agencies wishing to implement MTFC. All other authors declare no competing interests.

## Authors' contributions

LAP is the principal investigator of the Social Network Study. He collected the qualitative data, supervised the analysis of the qualitative data and collection and analysis of the survey data, and contributed substantially to the writing of the manuscript. IWH, ER, DF, and QW contributed substantially to data analysis and the writing of the manuscript. PC is the principal investigator of the parent study randomized trial that forms the basis for this study and contributed to the conceptualization, design, and writing of the manuscript. All authors read and approved the final manuscript.
